# Impacts of stigma and discrimination in the workplace on people living with psychosis

**DOI:** 10.1186/s12888-020-02614-z

**Published:** 2020-06-08

**Authors:** M. E. Hampson, B. D. Watt, R. E. Hicks

**Affiliations:** School of Psychology, Bond University, University Drive, Robina, Gold Coast, Queensland 4229 Australia

**Keywords:** Stigma, Discrimination, Employers, Workplace, Psychosis

## Abstract

**Background:**

Employment holds many benefits for people living with psychosis. However, significant barriers to employment for this cohort appear to exist, notably stigma and discrimination against people living with serious mental health conditions. We asked: Would a qualitative sample including multiple stakeholder groups reveal similar results and if so, what would be the main impacts of such stigma and discrimination?

**Method:**

This analysis used data from a qualitative study that had employed focus groups and interviews to investigate the employment barriers and support needs of people living with psychosis, including views of the multiple stakeholders (those living with mental health conditions, health professionals, care-givers, employments consultants and community members and employers).

**Results:**

The impacts of workplace stigma and discrimination on people living with psychosis included work avoidance, reluctance to disclose mental health conditions to employers, work-related stress, and reduced longevity of employment.

**Conclusions:**

Significant impacts from such stigma and discrimination were found in this study. The findings indicate a need to provide support mechanisms and to change the culture of workplaces to improve employment opportunities and outcomes for people living with psychosis.

## Background

Psychosis is a mental health condition characterised by symptoms including delusions, hallucinations, disorganised thinking and disorganised behaviour [1]. Psychotic symptoms, even when optimally treated, may persist and be extremely disruptive and impair ability to work. Morgan et al. [2] estimated the 12-month prevalence of psychosis among Australian residents aged 18–64 years in contact with public specialised mental health services at 4.5 persons per 1000 population. The study found that 32.7% of the sample had been in paid employment during the previous 12 months, a rate much lower than the general population. Research evidence indicates that stigma and discrimination are major barriers to employment for people living with psychosis (e.g. Schulze & Angermeyer [3]). It is important to study the impacts of work-related stigma and discrimination due to the important benefits of employment and significant costs of unemployment for people living with psychosis [4]. A large body of research underlines the personal, social and economic benefits of competitive employment for people living with psychosis, with benefits including clinical improvement [5] as well as quality of life benefits [4, 6]. Conversely, research highlights the significant personal, social and economic costs of unemployment among this cohort [4, 7]. Research has also shown that people living with psychosis have positive strengths to contribute to the workforce [8]. Recent studies also suggest there is growing recognition that people with lived experience of mental health conditions who are in the workforce contribute significantly to mental health service provision, and to research increasing our understanding of their abilities, responses and contributions [7–10].

This raises the question whether workplace leaders and managers can afford to allow stigma and discrimination in the workplace to persist, as it may make more business sense for workplaces to include people of diversity including people with lived experience of mental health conditions.

Perhaps the most famous studies on stigma towards people living with mental health problems have been those published by sociologist Goffman [11, 12]. The classic work of Goffman on the “stigmata”, “spoiled identity” and “labelling” of mental patients is no less relevant today than it was 50 years ago [11, 12]. Link and Phelan [13] suggest that stigma exists when elements of labelling, stereotyping, separation, status loss, and discrimination co-occur in a power situation that allows these processes to unfold. Empirical studies consistently confirm the ongoing phenomenon of public stigma towards people living with mental health problems. Research into public attitudes indicates strong negative stereotypes including perceptions that such people are unpredictable, aggressive, violent, dangerous, unreasonable, less intelligent, lacking in self-control and frightening [3, 14–16]. Studies of social distance indicate that few people would recommend a person living with schizophrenia for a job [3]. Mental health professionals have also been found to hold stigmatising attitudes towards their patients including negative perceptions concerning their employability [17–19]. Self-stigmatisation has been described as a process in which individuals internalise social stigma, with adverse impacts including diminished self-esteem and reduced self-efficacy [20]. The presence of stigma and discrimination in the workplace is affirmed by ongoing research on issues relating to disclosure of a mental health condition to an employer [21–23].

Corrigan and Penn [14] and Perkins et al. [15] found that integration of people with mental health conditions into gainful employment can play an important role in changing social attitudes and reducing the stigma associated with these conditions. Perkins et al. [15] pointed out that more research has focused on factors likely to increase social stigma towards people with psychiatric disabilities than on factors that de-stigmatise people living with these conditions. They administered a series of vignettes and a social distance rating scale to a sample of 404 adult interviewees and found that public attitudes were less stigmatizing towards people recovering from schizophrenia who were gainfully employed. Similarly, a survey by Hamilton et al. [23] found that employment may be a protective factor against the total level of discrimination experienced by people using mental health services in England. Schulz and Angermeyer [3] suggested that, in order to challenge the exclusion of people with schizophrenia from important life opportunities, it is particularly important to support them in the field of employment.

An extensive body of research identifies social stigma as a significant barrier to employment of people living with serious mental health conditions [24, 25]. Brohan et al. [26] reported related phenomena such as recruitment discrimination, misattribution of behaviours and stress surrounding disclosure of mental health problems in the workplace. In an Australian study Hampson, Hicks and Watt [27] found stigma and discrimination to be the most commonly referenced barrier to employment for people living with psychosis.

Previous studies have provided insights into key support needs of people living with psychosis. Peckham and Muller [25] identified employer education in mental health as an important support need. Henry and Lucca [24] identified reduction of social stigma as being among the most important barriers to the achievement of employment goals. McGahey et al. [21] found that a formal plan to support disclosure helps young job seekers with severe mental health problems to retain employment.

While public stigma and discrimination towards people with mental illness is widely recognised and there is a significant body of research on this topic, there is increasing interest in research about the origins and prevalence of mental illness stigma and discrimination in today’s workplace and about evidence-based best practice to overcome it [28]. International studies have identified high rates of discrimination towards people living with mental health issues [29, 30]. Recent research conducted in Australia has indicated continued high rates of actual and anticipated discrimination as well as concealment of a mental health condition due to anticipated discrimination [31], with other research indicating significant issues in male-dominated workforces [32]. Recent studies have also highlighted the need for support for workers with mental health issues around disclosure of their mental health status to an employer due to anticipated stigma and discrimination [21, 22]. Research suggests that employers prefer disclosure of a mental health condition by employees [32, 33]. However, Brohan et al. [26] found employees with mental health problems need to exercise care in disclosing a mental health condition in the employment context to avoid prejudice. They found inter alia that stigma and discrimination towards people with mental health problems could result in non-hiring and misattribution of workplace behaviours. Characteristics of the line manager and workplace culture were important factors in mediating disclosure.

In Australia there is increasing awareness of the personal, social and economic costs of stigma and discrimination towards mental health conditions [2, 4, 7, 31, 32]. There remains, however, limited knowledge regarding contemporary expressions and impacts of mental health stigma and discrimination in the workplace. The current paper uses data from a large qualitative study of employment barriers and support needs in psychosis to explore contemporary expressions and impacts of stigma and discrimination in the Australian workplace.

## Method

A qualitative approach was used to explore perceptions of people living with psychosis and other key stakeholder groups towards barriers to employment for people living with psychosis.

### Participants

A purposive sample of 137 participants was invited to take part in focus groups and/or individual interviews designed to elicit their perceptions of the employment barriers and employment-related support needs of people living with psychosis. The sample included participants from six key stakeholder groups: people living with psychosis (*n* = 25), care-givers (*n* = 9), employers (*n* = 11), health professionals (*n* = 19), employment consultants (*n* = 27), and community members (*n* = 46). Participants from multiple key stakeholder groups were included in the sample to gain a comprehensive understanding of the employment barriers and support needs of people living with psychosis. The purposive sample aimed to include people living with psychosis who were employed at the time of the study. The sub-sample of 25 people living with psychosis included those who were in paid employment at the time of the study (*n* = 9), as well as some client participants who, although they were not in paid employment at the time of the study, reported during focus groups or interviews that they had been employed previously. Basic demographic data were obtained from all participants. The sample included participants aged 18–84 years, reflecting a broad range of educational attainment and diverse occupational groupings. The researchers specifically sought to recruit interviewees whose employment experiences diverged from the norm: for example, employers with success employing people with psychosis, as well as individuals considered rich sources of data due to their life experiences.

### Procedure

Participants were recruited by approaching individuals, service providers and community organisations in South East Queensland. Ethical approval for this study was obtained from Bond University Human Research Ethics Committee. Participants were required to provide informed written consent. Only one prospective participant, who was acutely unwell at the time of the study, was excluded from the study.

Data collection proceeded in two stages: Fourteen focus groups were conducted followed by 31 individual interviews. Focus groups were used as they reduce the power differential between researcher and participants, allowing the voices of marginalised groups to be heard and providing relevant data for thematic analysis [34–37].

^.^ Individual interviews were conducted to broaden the range of individuals included in the study; capture new insights; include exceptional cases; address residual gaps in understanding; and test novel ideas raised in focus groups. Focus groups consisted of three to ten participants and comprised participants from the same stakeholder group to optimise freedom of expression.

Focus groups and interviews were conducted by a registered clinical psychologist, who provided ground rules for the group, minimal encouragers to promote expression of views, and prompts to keep the discussion on track. A second registered psychologist was present during client focus groups to provide support if needed to ensure client safety. The following two questions were posed to all focus groups to elicit participants’ perceptions of employment barriers and support needs of people living with psychosis:

- Question 1: We know that many people who have been diagnosed with schizophrenia or bipolar disorder would like to work in regular paid employment. We also know that the employment rate of people with these conditions is significantly lower than the general population. Why do you think this is the case?
- Question 2: What do you think would need to change in order to improve employment outcomes for people who have been diagnosed with schizophrenia or bipolar disorder?

Bipolar disorder and schizophrenia were used as examples of psychotic conditions as it was considered most people would have some knowledge and/or have heard about these conditions. There was no attempt in this study to compare responses between participants with different diagnoses.

Semi-structured interviews were constructed and applied during individual interviews, using a responsive interviewing style consistent with the approach described by Rubin and Rubin [35]. The questions posed to focus groups and interviewees did not specifically enquire about stigma and discrimination, thereby ensuring that participants were not primed to specify stigma and discrimination, but instead these issues were identified via open-ended questions. Focus groups and interviews continued until a point of saturation was reached when no new themes emerged^36.^ The data were analysed using thematic analysis [37]. The overall findings in relation to the employment barriers and support needs of people living with psychosis (that stigma and discrimination were the main barriers to employment) were set out in an earlier paper by Hampson, Hicks, and Watt [27]. The current paper focuses specifically on participants’ perceptions of the impacts of stigma and discrimination in the workplace on people living with psychosis.

Transcripts were thoroughly and repeatedly searched for all references to stigma and discrimination. References to stigma and discrimination were initially coded to free nodes or themes. For purposes of coding, the stigma node included references to “generalised negative attitudes, beliefs, perceptions and emotional responses to people living with a serious mental health condition”. The discrimination node included references to “actions and behaviours which demean and disadvantage people living with a serious mental health condition”. Through a process of thematic analysis, the stigma and discrimination nodes were then categorised into lower order nodes including nodes relating to the presence and impacts of stigma and discrimination in the workplace. The contents of each of these lower order nodes was explored in-depth to identify subordinate themes pertaining to the impacts of stigma and discrimination in the workplace. Further information concerning the composition of the sample and research design can be obtained by reference to Hampson [38].

In the following section, we present participant perceptions of stigma and discrimination in the workplace in relation to people living with psychosis. In the results section that follows, the main themes are presented and illustrated using relevant participant quotes.

## Results

The results indicated that stigma and discrimination have far-reaching effects on jobseekers and employees living with psychosis. Impacts affect many aspects of the employment experience including job-seeking, recruitment, workplace relationships, workplace communication and emotional well-being of employees. Impacts in each of these areas are described in more detail in the sections that follow.

### Impacts on job-seeking

Participants reported diverse impacts of stigma and discrimination on job-seeking including general work avoidance and avoidance of employment support services.

Some people living with psychosis were perceived to avoid job-seeking due to the possible need to disclose their condition to an employer and potential impacts of stigma and discrimination. Participants suggested that, due to self-stigmatisation and/or past negative experiences, some people living with psychosis believe others will not understand them and expect to be judged if they enter the workforce or return to work following a relapse. There was evidence that some people avoid work altogether due to fear of workplace rejection, preferring to associate with others in a similar situation. For example, a peer support worker commented:

A lot of people are afraid of people with schizophrenia too so they’re afraid of that rejection, you know. When they go places, if someone knows that person’s got schizophrenia then the other people in the workplace are gonna [sic] be scared of them, don’t want them there...and that is a real thing that still a lot of people don’t want those type of people around....so people just think, well, it’s easier to stick with staying home or hanging out with friends who also have schizophrenia....

Participants pointed out that some people choose not to use employment services due to concerns about disclosure, labelling and stigma. Others seemed to try to avoid stigma by seeking work independently without accessing government funded employment support programs. A mental health case manager remarked:

...a lot of people want to return back to the workplace but they want to do it on their own because they don’t want to be labelled with a mental illness going into the workplace. They’ve still got that stigma. They believe there’s that stigma still there, so they’ll attempt [to find work] themselves and probably do quite a poor job of trying to get back in the workplace.

### Impacts during the recruitment process

Stigma and discrimination were perceived to influence recruitment practices. A community member referred to a tendency for employers to avoid employing people known to have mental health problems:

You’re acting as being a business, you’re running a business and you’re gonna have ten people come up to ya [you], right, nine of them perfect and one of them got this problem [psychosis]. You gonna hire that one person? Yeah but I’m not saying that why, we’re saying what people don’t hire ‘em for. You know I’m not saying it’s right or wrong it’s what they do.

A number of participants alluded to attempts on the part of employers to screen out applicants with mental health conditions during the interview process. One participant who had been offered a job placement found that after she disclosed having bipolar disorder a job offer was not followed up. She recalled, “I could see body language change when I told them I was a bit bipolar ...”

### Impacts on workplace relationships

#### Treated differently

Participants reported being viewed and treated differently from other employees in the workplace. An interviewee living with bipolar disorder described being a victim of heightened scrutiny and baiting:

… after I’ve got the job they watch me like a hawk. And I’ve found a few jobs they actually bait me to see how I’ll go, whether I’ll go one way or the other. They’ll bait me to see what I’ll do… then she’d smile at me but she’d know it’d piss me off so she’d watch me react and unfortunately she got a reaction out of me and that’s how I lost one of my jobs.

.... people just sort of look at you and think you’re a little bit different, start treating you a little bit differently, watch you a little bit more closely.

This respondent also described a phenomenon relating to misattribution of her moods.

You can’t have happy days...like everyone has emotions; everybody has a happy day; everyone has a sad day but when you have bipolar it’s like ah no have you checked your medication lately? you’re really not quite well ...it’s like shit… no, I’m just having an off day like everybody else. Usually [it’s]when you’re having a disagreement with them that’s when something [like this] …comes up.

#### Less tolerance in the workplace

An experienced employment consultant expressed the view that some employers may be less tolerant of work absences if the employee is known to have a mental health condition:

The other aspect too with employers is that ‘I’ve tried one of those before’. You know if somebody has a mental illness and it doesn’t work out, some employers say ah been down that track and it doesn’t work, they’re unreliable; yet if a non-disabled person comes to Friday night and has a night on the tiles and doesn’t come to work on Saturday morning that’s normal behaviour for young people here on [XXX].

A community member expressed a similar view:

Do you think there’d be less tolerance? Like the employer gives someone a go and says alright I’m not going to put them in the box of ‘nutter.’ I’m going to give this person a go. He gives them a go and then he has an episode…he doesn’t turn up for work 1 day or he comes in and … his condition is affecting his performance and he misses a day’s work. Do you think that employer… may be less tolerant of the fact that he has had this episode rather than the person who wakes up in the morning and says, ‘Ah I’ve got a cold and I can’t come in’? You know would you let them get away with that but the other guy I can’t come in because I’m having an episode? And do you think the employer would say ‘I knew this would happen’?... Not cut him as much slack as the bludger who just can’t be bothered to get up that day?

In similar vein, a person with lived experience of bipolar disorder recalled, “I did give them [employer] a letter from my doctor and information around the illness and that I just needed some time off, but they just wouldn’t accept it.”

#### Victimisation

Victimisation in the workplace was perceived to take a variety of forms including rejection, bullying and harassment, humiliation, exploitation and unfair dismissal. Participants described being victims of ostracism, teasing and bullying. For example, a respondent living with schizophrenia said, “Work mates... they know you got a disability, they take it out on ya [you] and they pay out on ya [you] and so you just don’t want to be there”.

A community member pointed out that co-workers may feel uncomfortable, be resistant or reject such a person:

She’s been there [in the workplace] about 4 months and still the people [co-workers] tease her and they’re all young. Everyone’s only twenty and they don’t talk to her and include her and it’s sort of mean in the workplace but the employees just don’t mesh well with her. They just don’t want to have anything to do with them because they’re so different. Like this lady once tried to interact but some people just laughed at her and walked away from her and that puts the boss in a position because he has to say, “Okay, well you can’t treat her like that”. And that makes the boss look bad, and the employees get grumpy because they like teasing each other...

A client respondent referred to having been the victim of name-calling in the workplace and suggested this was more likely to occur following a disagreement with a co-worker:

For years I self-mutilated so my arms are full of scars... so you get called ‘slashy’ or ‘slasher’ or something like that…just names and that’s well that’s just part of it. You just let it go.

#### Inequitable remuneration and reduced opportunities for advancement

Participants cited inequitable employment practices including exploitative remuneration rates in sheltered workshops and lower remuneration rates in competitive employment situations. For example, a person who had been diagnosed with schizophrenia said:

I was getting one dollar per hour. And then after 2 weeks they said to me “What do you think of the job?” I said, “It’s horrible”. I said, “I’ve been to university and you’ve given me one dollar an hour for doing this”. I said, “I don’t want anything to do with it.”

There was a perception that if people living with psychosis do get jobs, they tend to be lower level jobs that are mismatched to their abilities and interests, and therefore not conducive to motivation or longevity of employment. For example, a peer support worker living with bipolar disorder commented:

Though I didn’t finish my degree and people say, ‘Ah, it’s good honest work’... I don’t want...I’m not going to go from studying science at university to being a check-out chick so there’s a lot of people like that, that’s very intelligent people but... their education was disrupted early...

### Impacts on communication

#### Taboo subject

Participants pointed out that communication can be difficult, as mental health problems still tend to be regarded as a taboo subject in the workplace. A tradesman living with bipolar disorder remarked:

Well from my point of view, from my experience, say if I [needed time off because I] was changing medications or stuff like that, communicating that with the boss.... it’s like a hard taboo subject…to talk about it...and if you do talk about it, you say “Ah look I’m changing medications” and then they go “Why” and you go “Ah well it’s because of this” and they don’t understand.... it’s hard to explain to someone, you know, I can’t function [while I’m changing my medication].

#### Disclosure difficulties

Participants reported some workers tend to be secretive about their condition and either do not disclose or partially disclose a condition in the workplace. Several participants were of the view that external social stigma and internalised self-stigma contribute to disclosure difficulties in the workplace. Disclosure difficulties can in turn impact on job retention and sustainability of employment. For example, a respondent living with bipolar disorder reported difficulty communicating with her employer due to a sense of shame associated with having a mental health condition :

I did have a really good job years ago and because of some of my behaviour at the time [related to mental health condition] I lost the job and my father wanted me to take it further and I wouldn’t just ‘cause of my shame around my illness but I probably should have pursued that but there was no support then to return to work. And…once again, you’re dealing with someone that’s been really unwell that doesn’t have that confidence to pursue that with the employer, to have that conversation, and probably they’re ashamed of their illness and they will leave rather than return to work.

Another participant pointed out that people may conceal their condition from work colleagues due to concerns about the consequences of disclosure:

I know a young woman who has serious bipolar disorder...but she understands it, she takes her medication and you and I could meet her in the workplace and you would have *no* idea... whilst working with her, I never dreamed that she had bipolar. We had never discussed it. There was never any opportunity. She certainly didn’t talk widely about it. It was only after my son’s incident that she came to me and said, you know, “I’d like to have a talk to you” and she said “I don’t tell anyone because”, she said, “straight away, it’s like there’s a barrier that people don’t understand or are frightened of it” ...she said, “I try not to tell anyone”. I think at that stage her employer did not know....that she was quite heavily medicated but well in control, just absolutely, so she had never felt comfortable enough to tell anyone... and it’s only that she shared it and I felt really sorry for her then...I thought that’s sad that you have to hide that sort of thing…

Another participant reported difficulty explaining his need to attend appointments and take medication at work:

… it’s hard when, you know, like you’re taking medication and someone sees you and they ask you what it’s for or you have to go “Ah I’m going to the doctor”... all the time (laughs) or I’m going for a..... yeah, they start asking questions so that’s kind of hard…

Employment consultants, in discussing the dilemma of whether or not to disclose a mental health condition to an employer, remarked:

A lot of it also comes down to whether they disclose [having a mental health condition]. A lot of times we have that argument on our hands with new clients. Do I disclose [or] do I not? You’re sort of damned if you do and you’re damned if you don’t. Do I let those barriers down and tell the person [employer] and be honest because I may need to have periods off? Will that employer be willing to give me that time, or am I cutting my nose off to spite my face by telling them because I may not need any of that time off? So I tell them and I might not get the job but if I don’t tell them and I do need that time off they’re not gonna be aware of it and at least if they do know there may be a little bit of leniency there but that could also backfire on me and not get me the job.

Non-disclosure may also affect employment consultants’ willingness to assist jobseekers, with some employment service providers regarding disclosure as crucial to a successful employment outcome. For example, one specialist disability employment consultant commented:

... in terms of helping and facilitating a good job match and supporting someone through an employment placement, disclosure is imperative. If you don’t have disclosure.....we can [only] point them in the direction of the job. We can assist with interview techniques and all those things that build around placement however we can’t help ‘em any further than that.

Non-disclosure to an employer and/or to co-workers, due to fear of stigma and discrimination, may also result in a lack of employment support in the event of a relapse. Conversely having a supportive employer facilitates disclosure. A participant living with bipolar disorder commented:

Some [employers] are supportive, some aren’t. The one I’ve got at the moment is very, very supportive. It depends if they’re a supportive boss. If they’re supportive and you know they’re gonna be supportive give them a hundred percent [disclosure], don’t worry about it but if they’re not supportive no don’t tell them a damn thing ‘cause they’ll hold it against you.

### Impacts on emotional wellbeing

Responses indicated that stigma and discrimination contribute to work-related stress through several mechanisms. The need to conceal a condition from employers and co-workers can itself generate increased work-related stress. A major theme raised by participants was the stress generated by the decisions around disclosure of a mental health condition to an employer due to the significant risks of disclosure or benefits of non-disclosure. Table 1 lists benefits and costs of disclosure and non-disclosure, as perceived by participants.

**Table 1:** Cost-Benefit Analysis of Disclosure in Psychosis

Benefits of Disclosure	Benefits of Non-Disclosure
Would help the employer to understand unexplained absences from work Access to provision of employment support including ESP and employer support Employer empathy and understanding; leniency if time is needed off work Co-workers may be more understanding Personal preference to be upfront Promotes sustainable work-through enabling employer education as well as contact and communication between employer and case managers if needed Would promote attitudinal change Relief at not having to fabricate reasons for attendance at appointments.	Improved chances of gaining interviews and securing employment Greater need to extend yourself which may help to build resilience. “…*if you don’t disclose then you tend to push through that a bit more and stretch yourself.”*
**Costs of Disclosure**	**Costs of Non-Disclosure**
Stigma-people look at you and think you are a little bit different; categorisation, labelling, stereotyping (preconceived ideas), being judged, changed perceptions (de-valued, less respect) Discrimination- restricts employment opportunities (ability to secure interview, gain jobs); risks of being viewed and treated differently in the workplace including coming under closer surveillance and being subject to bullying (name-calling, baiting, loss of employment); loss of business “*And then I find after I’ve got the job they watch me like a hawk. And I’ve found a few jobs they actually bait me to see how I’ll go…”* Negative reaction from employer-behaviour attracts closer scrutiny from employer; employer may look for problems; misinterpretation of behaviour and moods, perception that they need extra support and are harder to accommodate People have less regard for you or hold you at a distance Can affect social relationships in the workplace-can affect acceptance by co-workers (concerns about contagion), difficulty making friends. Avoidance. Rejection (due to fear). People “hold you at a distance”. Separation. Self-victimisation “*…if you do disclose, I think it’s too easy just to become like a victim...I’ve got this condition, I’m not feeling too good, I’ll have a day off.”* Compromise professional registration Inability to control extent of disclosure within the workplace including how many people “need to know”. Not knowing what to divulge and what not to and how this information may be used against you later	Requires additional effort to fill unexplained gaps in resumé Increased pressure due to need to maintain constant state of vigilance to guard secrecy and hide the condition from others; need to prove you are as good as everybody else; difficulty explaining treatment needs; difficulty explaining inability to function; fear of inability to sustain work or relapse requiring time off work Unexplained “drugged out” appearance Employer unaware of need for empathy and support - may affect sustainability of employment Employer less sympathetic in event of needing time off work or support to return to work following relapse- affects sustainability of work. Precludes informal collegial support. Inability to access relevant employment support, particularly during periods of exacerbation or relapse Employer unprepared to deal with situation appropriately Employer may mistakenly think they are lazy, unreliable or refuse to give a reference Perpetuation of stereotypes and lack of opportunity to challenge prevailing attitudes Unsuitable or unsustainable work -setting themselves up to fail, exploitation Dismissal for false declaration

Disclosure was commonly associated with fear, ambivalence, discomfort and embarrassment. Disclosure was also seen to carry the risk of discrimination, including various forms of victimization and heightened scrutiny in the workplace. A client participant said, “I’d rather tell them that right up front because I’ve always been upfront...that I am bipolar but I feel you get discriminated against...it happened to me”. Conversely non-disclosure to an employer was perceived to have stressful consequences for the employee. Importantly, it was pointed out that non-disclosure would preclude access to appropriate employment support if required.

The need to be secretive and conceal a condition from employers and co-workers was reported to create moral dilemmas. A participant with lived experience of bipolar disorder said, “I’ve found I need to be a little bit more cautious about who I tell-*although I’m a woman of integrity-* because some people judge you”. Others, who chose not to disclose, felt they had to resort to fabrication to explain absences:

Interviewer: how did you feel after disclosing it [mental health condition] to her [employer]?

Respondent: Um a bit of relief because I didn’t have to (loud sigh), not make up stories, but just sort of fabricate things and where I’m, you know...can I take this day off I’ve got an appointment and she’s like great what’s this appointment? She’d never ask me what the appointments were but in a way I kind of felt it was my duty to tell her.

One participant acknowledged he had resorted to deception to fill gaps in his resume saying, “I’m a good con artist when it comes to writing job applications. I’m a con artist, yeah… when it comes to that…I know how to present at interviews”.

A participant with lived experience pointed out that non-disclosure heightens the ongoing fear of relapse due to anticipated adverse reactions to relapse from their employer and colleagues who may be unaware of or lack understanding of these conditions.

The pressure to prove oneself to be as good as others in the workplace was also identified. A community participant, having discovered a work colleague was living with bipolar disorder, reflected:

... I thought you’ve lived with this [bipolar disorder] for years, trying to do all the right things, trying to prove that you’re as good as anybody else and you are*,* but for some reason she’d felt she had to do a better job. She had to make sure that everyone knew she was as good as everybody else. I thought that’s sad that you have to bend over backwards to prove that you’re as good as everybody else when there’s nothing to prove. She was excellent. She was a wonderful employee and is.

These stressors were seen to contribute to exacerbation of symptoms, decreased ability to cope and reduced capacity to sustain employment. The data indicates some non-disclosing clients, when unable to sustain work, would prefer to simply leave their employment rather than disclose their condition to an employer.

It should be pointed out several participants living with psychosis reported having had supportive employers and positive workplace experiences. Other participants pointed to the potential of people living with psychosis to become excellent employees. Importantly, many participants expressed the need for culture change in the workplace to overcome stigma towards people living with psychosis and to give them a “fair go”.

## Discussion

Although discrimination in the workplace is illegal in Australia, responses in this study indicate that stigma and discrimination were perceived to persist in the workplace. The current prevalence of mental health stigma and discrimination in the Australian workplace is unknown and there are challenges in relation to how stigma and its prevalence can best be measured.

**Fig. 1:**
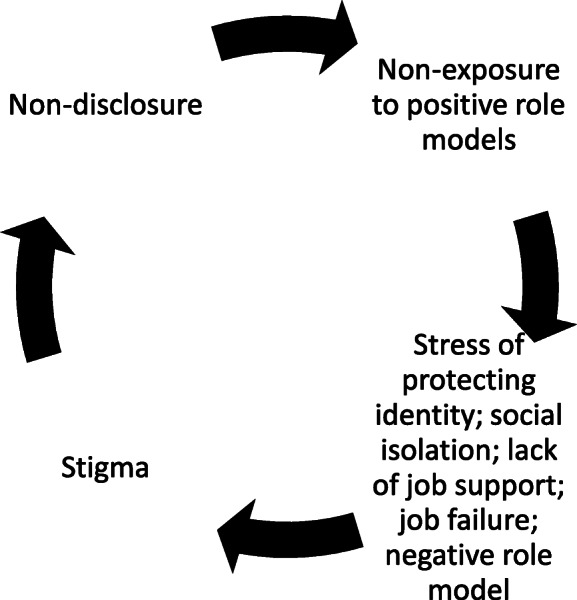
Self-perpetuating cycle of stigma

This study points to some of the far-reaching personal, social and economic consequences of mental health stigma in the workplace for people living with psychosis. The many and diverse impacts not only predispose people to job failure but contribute to a self-perpetuating cycle of stigma (see Fig. 1).

The results of this study confirm the findings of Brohan et al. [26] regarding personal impacts of labelling in the workplace including recruitment discrimination, misattribution of workplace behaviours and difficulty associated with disclosure or concealment of mental health problems. It also supports their finding of the important roles played by the employer and workplace culture in disclosure.

The findings of this study confirm that stigma and discrimination are significant barriers to employment and significantly affect the employment experiences of people living with psychosis. The findings of this study also support the findings of Brohan et al. [26] that stigma and discrimination impact all aspects of employment including recruitment, workplace relationships and workplace wellbeing, and significantly affect individuals’ ability to obtain and maintain employment. The importance of this study is that it demonstrates contemporary expressions of stigma and discrimination in the context of employment of Australians living with psychosis.

A limitation of this study is that the main research questions were designed to explore employment barriers and support needs of people living with psychosis. Future studies should design research questions more specifically targeted to investigate employment-related stigma and discrimination.

Stigma and discrimination in the workplace towards people living with psychosis have many adverse personal, social and economic consequences for the individuals involved as well as the wider community. The impacts of unemployment on this group and their families are devastating and far-reaching, affecting their health and well-being and compounding their sense of social isolation and marginalisation. It is important that workplace stigma and discrimination be addressed and overcome to enable more people living with psychosis to sustain employment because employment itself appears to be an important factor in de-stigmatising individuals living with mental health problems in the broader society [15].

The origins of the mental health stigma and discrimination remain obscure [13]; however, health, welfare and economic imperatives demand that more be done to overcome employment-related stigma and discrimination towards people living with psychosis.

In Australia, in addition to anti-discrimination legislation, there is government-funded research in progress to explore effective strategies to reduce stigma and discrimination in the workplace; anti-stigma campaigns, and mental health literacy courses. However, much remains to be done to educate employers and co-workers, allay their fears and improve their capacity to make reasonable workplace adjustments and respond appropriately to situations that may arise in the workplace. A strong argument could be made for more funding to educate employers and the public concerning more severe mental health conditions such as psychosis, its potential impacts on those affected and how best employers can support people living with these conditions. Early education in schools is needed to challenge negative stereotypes enabling more opportunities and support for people living with psychosis to participate in the workforce [39]. There are also implications for professionals assisting people living with psychosis to anticipate and rehearse strategies to address stigma and discrimination, including discussion around pros and cons of disclosure [21, 31, 32]. The findings support the need for more widespread use of evidence-based decision aids to assist in disclosure of mental health status to an employer such as the one developed by Henderson, Brohan et al. [40], as well as the development of evidence-based resources for employers and employees to help reduce the impacts of stigma and discrimination in the workplace [41].

While the focus of this paper was on the expression and impacts of stigma and discrimination in the workplace for people living with psychosis, it is important to note that some respondents in this study reported positive experiences in relation to their workplaces and employers, indicating that more positive attitudes and behaviours in the workplace towards employees living with psychosis are achievable. Overcoming stigma and discrimination towards people living with psychosis constitutes a major challenge to employers striving to achieve the social and economic benefits of a diverse and inclusive workforce.

## Conclusions

Our study has confirmed that there are significant impacts of workplace stigma and discrimination on those living with psychosis. While many organisations have opened their organisations more widely and demonstrate inclusive supportive behaviour for diverse groups including those with mental health disabilities, there is strong evidence from our study that much more needs to happen. The findings indicate a need for employers, health professionals, and policy makers to think clearly about the value to our society and to the individuals with lived personal experience of psychosis of including such individuals within the workforce. The findings of our study indicate the need to change the culture of workplaces and the attitudes towards social inclusion of diverse groups, to improve employment opportunities and outcomes for people living with psychosis. Our health professionals and employers contribute substantially in this area through understanding and supporting the inclusion within the workforce of such individuals who can contribute soundly to organisations and develop their own mental wellbeing through social inclusion.

## Data Availability

The data is stored under e-password protected files with the Primary Supervisor (the corresponding author) under University rules; the data is also stored with the Lead author who conducted the primary research. The authors declare that the data may be made available on request through the corresponding author.
